# Resistance wheel running improves contractile strength, but not metabolic capacity, in a murine model of volumetric muscle loss injury

**DOI:** 10.1113/EP091284

**Published:** 2023-08-10

**Authors:** Albino G. Schifino, Christiana J. Raymond‐Pope, Junwon Heo, Jennifer McFaline‐Figueroa, Jarrod A. Call, Sarah M. Greising

**Affiliations:** ^1^ Department of Kinesiology University of Georgia Athens GA USA; ^2^ School of Kinesiology University of Minnesota Minneapolis MN USA; ^3^ Department of Physiology and Pharmacology University of Georgia Athens GA USA; ^4^ Regenerative Bioscience Center University of Georgia Athens GA USA

**Keywords:** CK clamp, neuromusculoskeletal injury, rehabilitation, skeletal muscle injury

## Abstract

The primary objective of this study was to determine if low‐ or high‐resistance voluntary wheel running leads to functional improvements in muscle strength (i.e., isometric and isokinetic torque) and metabolic function (i.e., permeabilized fibre bundle mitochondrial respiration) after a volumetric muscle loss (VML) injury. C57BL/6J mice were randomized into one of four experimental groups at age 12 weeks: uninjured control, VML untreated (VML), low‐resistance wheel running (VML‐LR) and high‐resistance wheel running (VML‐HR). All mice, excluding the uninjured, were subject to a unilateral VML injury to the plantar flexor muscles and wheel running began 3 days post‐VML. At 8 weeks post‐VML, peak isometric torque was greater in uninjured compared to all VML‐injured groups, but both VML‐LR and VML‐HR had greater (∼32%) peak isometric torque compared to VML. All VML‐injured groups had less isokinetic torque compared to uninjured, and there was no statistical difference among VML, VML‐LR and VML‐HR. No differences in cumulative running distance were observed between VML‐LR and VML‐HR groups. Because adaptations in VML‐HR peak isometric torque were attributed to greater gastrocnemius muscle mass, atrophy‐ and hypertrophy‐related protein content and post‐translational modifications were explored via immunoblot; however, results were inconclusive. Permeabilized fibre bundle mitochondrial oxygen consumption was 22% greater in uninjured compared to VML, but there was no statistical difference among VML, VML‐LR and VML‐HR. Furthermore, neither wheel running group demonstrated a change in the relative protein content of the mitochondrial biogenesis transcription factor, peroxisome proliferator‐activated receptor γ coactivator 1‐α (PGC‐1α). These results indicate that resistance wheel running alone only has modest benefits in the VML‐injured muscle.

## Abstract (highlights)

- **What is the central question of the study?**
  Does initiation of a resistance wheel running regimen following volumetric muscle loss (VML) improve the functional capacity of skeletal muscle?
- **What is the main finding and its importance?**
  Resistance wheel running led to greater muscle mass and strength in mice with a VML injury but did not result in a full recovery. Neither low‐ nor high‐resistance wheel running was associated with a change in permeabilized muscle fibre respiration despite runners having greater whole‐body treadmill endurance capacity, suggesting resilience to metabolic adaptations in VML‐injured muscle. Resistance wheel running may be a suitable adjuvant rehabilitation strategy, but alone does not fully mitigate VML pathology.

## 1 INTRODUCTION

Skeletal muscle is a highly plastic tissue that adapts to changing metabolic and contractile demands when placed under stress, such as overload that occurs during exercise. Consistent, progressive exercise elicits various positive adaptations depending on the type of stimulus/overload applied, through the process of mechanotransduction (Dunn & Olmedo, 2016). Adaptations resulting from resistance exercise include increased muscle size through contractile protein synthesis, satellite cell proliferation, motor unit recruitment, and increased contractile force. When the stimulus applied is an endurance‐type overload, the oxidative and metabolic capacity of the muscle is improved through enhanced mitochondrial density and function, capillary density and activity of oxidative enzymes, ultimately improving oxygen use efficiency (Joyner & Coyle, 2008). These local muscle adaptations result from stimulation of various intracellular signalling pathways, such as the phosphoinositide 3‐kinase (PI3K)/Akt/mechanistic target of rapamycin (mTOR) pathway for protein synthesis, and downstream targets, such as the mitochondrial biogenesis regulator peroxisome proliferator‐activated receptor γ coactivator 1‐α (PGC‐1α), and activation of enzymes involved in mitochondrial function. Improvements in whole‐body functions (e.g., contractile, metabolic, cardiorespiratory) will occur concurrent with these local muscle adaptations in a manner dependent on the type of exercises implemented (e.g., resistance or aerobic). In combination, resistance and aerobic type exercises are paramount for eliciting optimal muscle and whole‐body function to mitigate age‐ or disease‐related decline.

For many recoverable musculoskeletal injuries (e.g., muscle strain, anterior cruciate ligament (ACL) injury), progressive overload in resistance exercise and aerobic training is often prescribed as part of evidence‐based guidelines for physical rehabilitation (Järvinen et al., 2007). The physical rehabilitation process, which involves multiple stages varying in length of time and exercise type, includes planned exercise as a stimulus to facilitate physiologically beneficial adaptations through progressive overloading of the afflicted body region, or in some cases, the whole body, to restore function (Greising et al., 2020; Myer et al., 2006). Often, as in the case of ACL rehabilitation, body weight and resistance exercises targeted to the affected muscle groups (e.g., quadriceps muscle group) are employed in the initial stages of physical rehabilitation to enhance the muscle's force‐producing capacity. Prior to returning to full activity, a running programme with agility is added to enhance the mobility of the joint and improve the oxidative capacity of the muscle and the whole body. Progressive overloading and aerobic exercise following injury often result in similar adaptations observed in healthy skeletal muscle, including increased muscle size and strength (Gerber et al., 2009). However, following severe, complex musculoskeletal injuries, such as a volumetric muscle loss (VML) injury in which a large irrecoverable volume of muscle is lost due to traumatic injury or surgical removal (Garg et al., 2015; Grogan & Hsu, 2011), no physical rehabilitation protocol has achieved complete recovery of muscle strength.

Currently, no standardized clinical physical rehabilitation regimen exists to treat VML injury, and physical rehabilitation may be delayed due to complications occurring immediately following initial treatment (e.g., debridement, muscle flap transfer). Identifying clinically translatable physical rehabilitation strategies that address the lack of regeneration and contractile dysfunction chronically following VML is needed. In animal models of VML injury, several physical rehabilitation strategies following injury have been tested (e.g., forced treadmill running, running/walking, intermittent electrical nerve stimulation), varying in type, frequency, volume, and intensity. One of the most common physical rehabilitation modalities employed preclinically is voluntary wheel running, beginning up to 1 week following VML injury (Aurora et al., 2014, 2015; Corona et al., 2013; Greising et al., 2018; Nakayama et al., 2018; Quarta et al., 2017; Southern et al., 2017; Washington et al., 2021). Wheel running is beneficial as the training is self‐selected and the volume of running is generally greater than with forced treadmill running (Lerman et al., 2002). Although some reports note wheel running following VML to promote muscle hypertrophy and strength and to attenuate inflammation compared to no physical rehabilitation (Aurora et al., 2014; Nakayama et al., 2018; Quarta et al., 2017; Washington et al., 2021), others have found no clinically significant improvements in these outcomes (Aurora et al., 2015). It is possible that a regimen that incorporates additional loading during wheel running bouts may enhance the force‐producing capacity of the muscle while facilitating increased muscle size and metabolic adaptations in the affected muscle.

Originally designed as an aerobic training paradigm, running wheels can be modified to add a load or weights to the circumference of the wheel, by fastening a manual tensioning device, or through servo‐motor‐generated resistance breaks. In animal models of disease (e.g., muscular dystrophy), ageing, and disuse, low‐ and high‐resistance wheel running regimens confer beneficial adaptations, including atrophy attenuation and muscle strength improvements (Call et al., 2010). Given the similar muscle pathology between diseases such as muscular dystrophy and following VML injury (Aguilar et al., 2018; Villalta et al., 2009), a physical rehabilitation strategy that implements a progressive resistance wheel running regimen may elicit similar adaptations within the muscle remaining. Additionally, a regimen that incorporates early physical rehabilitation (i.e., 3 days) following VML may improve long‐term outcomes. Indeed, clinical physical rehabilitation regimens have noted that early mobilization following recoverable musculoskeletal injury (e.g., ACL injury) improves functional outcomes; therefore, investigating the early initiation of progressive resistance wheel running following VML injury in a preclinical model is warranted.

The primary objective of this study was to evaluate skeletal muscle adaptations and functional capacity in response to a low‐ or high‐resistance wheel running regimen initiated early following VML injury. Further, the study aimed to evaluate optimal intensities of overload with a low‐ and high‐resistance protocol, with the hypothesis that 8 weeks of resistance wheel running exercise would improve skeletal muscle contractile and metabolic function and enhance skeletal muscle mass and that these adaptations would be greater with high‐resistance wheel running.

## 2 METHODS

### 2.1 Ethical approval

Male C57BL/6J mice (The Jackson Laboratory, Bar Harbor, ME, USA; stock no. 000664) were group‐housed with food and water provided ad libitum. Housing temperature was maintained at 20–23°C on a 12‐h light–dark cycle (07.00–14.00 h). Following 1 week of acclimatization, animals were randomly assigned to experimental groups. All protocols and animal care guidelines were approved by the Institutional Animal Care and Use Committee (A2020 06‐023). Procedures were performed in compliance with the Animal Welfare Act, and the Implementing Animal Welfare Regulations in accordance with the principles of the *Guide for the Care and Use of Laboratory Animals*.

### 2.2 Experimental design

Mice were randomized to one of four experimental groups at age 12 weeks: uninjured (*n* = 8), VML untreated (VML, *n* = 12), VML high‐resistance wheel running (VML‐HR, *n* = 8), and VML low‐resistance wheel running (VML‐LR, *n* = 8). Uninjured mice underwent no interventions until killing at 20 weeks of age. VML groups underwent a unilateral VML injury to the left hindlimb plantar flexor muscles (gastrocnemius, plantaris, soleus muscles) at age 12 weeks. At age 20 weeks, mice in all experimental groups performed an exhaustive treadmill capacity test to determine whole‐body aerobic adaptations. Three days following the exhaustive treadmill capacity test, all experimental groups were tested for in vivo muscle contractile function of the plantar flexor muscles. Immediately following functional measurements, mice were killed by CO_2_ inhalation and cervical dislocation. Hindlimb muscles were removed, weighed, and gastrocnemius muscle was subsequently prepped for ex vivo analysis of metabolic function by high‐resolution respirometry or snap‐frozen in liquid N_2_ for further analyses.

### 2.3 Volumetric muscle loss injury model

Unilateral VML injury to the left hindlimb plantar flexors (gastrocnemius, soleus, plantaris) provided volumetric removal of muscle tissue from anaesthetized (isoflurane 1.5−3.0%) mice, as previously described (Greising et al., 2018; Southern et al., 2019). Mice were administered buprenorphine (2.0 mg/kg; s.q.) prior to a single incision being made in the mid‐gastrocnemius from distal to proximal exposing posterior compartment muscles, then again at 24, 48 and 72 h post‐surgery. A 4 mm standardized biopsy punch was used to remove ∼15% (26.40 ± 2.50 mg) of muscle tissue. Any bleeding was stopped with light pressure. Skin incisions were closed with 6.0 silk sutures and mice were monitored through recovery.

### 2.4 Resistance wheel running protocol

Both VML‐HR and VML‐LR cohorts performed 8 weeks of voluntary wheel running on cage‐mounted wheels (Home Cage Running Wheel, Columbus Instruments, Columbus, OH, USA, cat. no. 43204), equipped with a digital sensor to monitor wheel counts. Additional weight (metal and epoxy) was affixed in a balanced manner (i.e., four weights equally distributed) to the exterior surface of the running wheel, and the masses (VML‐HR: 6 g; VML‐LR: 1 g) were selected based on existing resistance wheel literature (Call et al., 2010; Murach et al., 2020). All mice in the VML‐HR and VML‐LR groups were individually housed and provided free access to their wheels. The external load represented ∼20.5% and 3.5% of terminal body mass for the VML‐HR and VML‐LR runners, respectively.

### 2.5 Exhaustive treadmill capacity test

Each mouse was familiarized with the treadmill for 5 min at low speeds (2–5 m/min) for three consecutive days prior to the treadmill testing. On the day of the test, mice were placed on the treadmill (Columbus Instruments) in individual lanes. The treadmill protocol started with two stages, 7.5 m/min for 7 min and 10 m/min for 7 min followed by an increase of 2.5 m/min every 10 min until a maximum speed was achieved of 27.5 m/min (Okutsu et al., 2014; Southern et al., 2017). Percentage grade remained constant throughout the test at 5%. Brushes at the end of the treadmill lane encouraged mice to keep running throughout the test. The fatigue test was terminated when mice no longer responded to five consecutive, forceful taps with the brushes. Treadmill distance and time were recorded for all mice.

### 2.6 In vivo plantarflexion muscle function

Muscle contractile strength of the hindlimb plantar flexors was assessed at the terminal time point in anaesthetized mice using (1.5–2.0%) isoflurane, as previously described (Baltgalvis et al., 2012; McFalin et al., 2022; Raymond‐Pope et al., 2023). Briefly, the animal's foot was placed in a footplate attached to a servomotor (Model 300C‐LR; Aurora Scientific, Aurora, Ontario, Canada). With the ankle joint positioned at a 90° angle (defined as a neutral position 0°), contraction of the plantar flexors was controlled by stimulation of the sciatic nerve after the peroneal nerve had been severed to avoid recruitment of the antagonist dorsiflexor muscles. Peak isometric torque (millinewton metres, mN·m) was achieved by varying the voltage delivered to the sciatic nerve at a frequency of 150 Hz and a 0.1 ms square wave pulse. Next, for the torque–velocity relationship, the foot was passively moved to 20° dorsiflexion, and a concentric contraction was performed at 150 Hz with the foot simultaneously moving to 20° plantarflexion (40°) at a rate of 800°/s. This procedure was repeated with concentric contractions performed at 600°, 400°, 200°, 100°, and 0°/s. Isokinetic power (watts, W) was calculated as follows: first, the contractile velocity in °/s was converted to repetitions per minute (RPM) such that 100°/s equals 16.667 RPM and 200°/s equals 33.333 RPM and 800°/s equals 133.333 RPM; second, for each contractile velocity the product of RPM and torque (mN·m) was divided by 9.5488. Peak isokinetic power represents the absolute maximal power calculated for each animal that was recorded during the torque–velocity test.

### 2.7 Ex vivo permeabilized fibre bundle mitochondrial oxygen consumption via high‐resolution respirometry

High‐resolution oxygen respiration measurement was conducted on gastrocnemius samples using an Oroboros Oxygraph‐2K (Oroboros Instruments, Innsbruck, Austria) and the creatine kinase (CK) clamp technique as previously described (McFalin et al., 2023). Briefly, mitochondrial respiration ($J_{O_{2}}$) and electron conductance were tested by using a CK clamp system wherein the enzymatic reaction of CK and phosphocreatine (PCr) is leveraged to manipulate the levels of cellular energy demand (Gibbs free energy; Δ*G*_ATP_) (Fisher‐Wellman et al., 2018). The equilibrium constant of CK (*K′*_CK_) was utilized to calculate the free energy of ATP hydrolysis (Δ*G*_ATP_) in the presence of known properties of Cr, PCr and ATP through an online resource (https://dmpio.github.io/bioenergetic‐calculators/ck_clamp/) as previously mentioned (Fisher‐Wellman et al., 2018).

Briefly, following the dissection of the medial part of the gastrocnemius muscle, the tissues were weighed and dissociated using micro‐forceps. Subsequently, the tissues were permeabilized with saponin (50 μg/ml) and then placed into the respiration chambers (2 mg fibre bundles per chamber). Respiration of the permeabilized fibre bundle mitochondria was initiated by adding substrates (10 mM glutamate, 5 mM malate and 10 mM succinate) with 20 U/ml CK and 5 mM ATP, followed by sequential PCr titrations (1, 1, 2, 3, 9 and 15 mM) to reduce Δ*G*_ATP_ to resting conditions. Δ*G*_ATP_ was plotted against the corresponding $J_{O_{2}}$, showing a linear relationship where the slope indicates the conductance (efficiency of electron flow) throughout the electron transport system. The basal rate without substrates was subtracted from all other stages, and rates were normalized to the wet weight of tissue loaded into each respiration chamber and citrate synthase (CS) activity to account for differences in the mitochondrial content.

### 2.8 Citrate synthase activity

Mitochondrial content was analysed by CS activity, an indirect measure/estimate of mitochondrial content (Larsen et al., 2012), as previously described (McFalin et al., 2022; Southern et al., 2019), using remaining muscle fibre bundles isolated from the medial part of the gastrocnemius during preparation for the CK clamp respiration experiments.

### 2.9 Immunoblot analyses

For protein expression level analysis, the protein concentration of muscle homogenates was determined by analysing absorbance at 562 nm via a Spectrophotometer. Twenty microgram of total protein was loaded and separated by SDS‐PAGE, transferred to a polyvinylidene difluoride membrane and immunoblotted. The following primary antibodies were used: muscle ring‐finger protein‐1 (MuRF1; ECM Biosciences (Versailles, KY, USA) cat. no. MP3401; RRID: AB_2208832, 1:1000), atrogin‐1 (ECM Biosciences cat. no. AP2041; RRID:AB_2246979, 1:1000), PGC‐1α (Abcam (Waltham, MA, USA) cat. no. ab54481; RRID:AB_881987, 1:1000), Akt (Cell Signaling Technology (Danvers, MA, USA) cat. no. 9272; RRID:AB_329827, 1:1000), phospho‐Akt (Cell Signaling Technology cat. no. 9271; RRID:AB_329825, 1:1000), mTOR (Cell Signaling Technology cat. no. 2972; RRID:AB_330978. 1:1000), phospho‐mTOR (Cell Signaling Technology cat. no. 2971; RRID:AB_330970. 1:100), p70S6K (Cell Signaling Technology cat. no. 2708; RRID:AB_390722, 1:1000), and phospho‐p70S6K (Cell Signaling Technology cat. no. 9205; RRID:AB_330944, 1:1000). Primary antibodies were detected using a corresponding host‐ and isotype‐specific fluorescence conjugated secondary antibody, DyLight 800 (Thermo Fisher Scientific (Waltham, MA, USA), cat. no. SA5‐10036) or horseradish peroxidase‐conjugated secondary antibody (Bio‐Rad Laboratories (Hercules, CA, USA) cat. no. 1706515). Immunoblots were blocked with 5% BSA, and primary and secondary antibodies were diluted with 5% BSA. Immunoblots were visualized with stain‐free and fluorescence or chemiluminescence imaging using a ChemiDoc MP System (Bio‐Rad Laboratories) (Gürtler et al., 2013). Immunoblots imaged using chemiluminescence were first incubated with enhanced chemiluminescence substrate for 5 min prior to imaging (Bio‐Rad Laboratories cat. no. 1705061). Using the stain‐free image, the total protein in each lane was quantified (Collins et al., 2015; Vigelsø et al., 2015; Zeitler et al., 2016), and the band of interest was identified using the corresponding fluorescence or chemiluminescence image at the appropriate molecular mass according to the manufacturer's technical information. The intensity of each band of interest was normalized to the total lane protein in each respective lane using Image Lab software (Bio‐Rad Laboratories).

### 2.10 Statistical analyses

All data are represented as means ± standard deviation (SD). One‐way ANOVA was used to detect differences across the experimental groups for variables of body and gastrocnemius muscle mass, skeletal muscle function (e.g., peak isometric torque), running distance during an exhaustive treadmill capacity test, $J_{O_{2}}$ and protein content. Two‐way ANOVA was used to evaluate power output across contractile velocities, $J_{O_{2}}$ across clamped ∆*G*_ATP_, and average daily running distances across training weeks. In the event significant differences were detected, a Tukey's HSD *post hoc* test was performed to explore distinct group differences. The non‐parametric Kruskal–Wallis test was used to assess electron conductance because it failed the equal variance assumption of performing an ANOVA. All statistical analyses were performed using JMP statistical software (JMP, Version 16.0, SAS Institute Inc., Cary, NC, USA), and all graphs were constructed using Prism (GraphPad Software (San Diego, CA, USA), version 9.4.1 for Windows).

## 3 RESULTS

**FIGURE 1:**
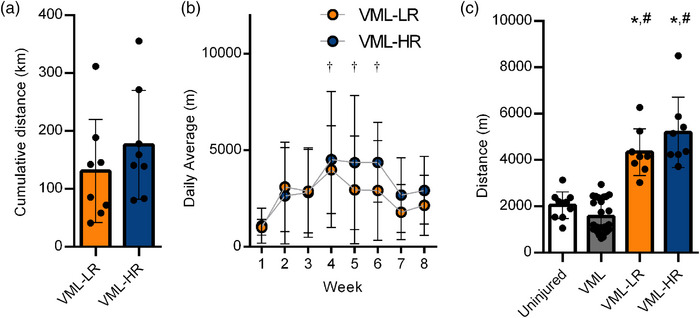
Effect of resistance on average daily wheel running distance and whole‐body endurance capacity. (a) Cumulative voluntary wheel running distance over 8 weeks for low‐ (LR) and high‐ (HR) resistance wheel runners following a VML injury. Results analysed using a unpaired Student's *t*‐test. (b) Average daily running distance per week for LR and HR wheel runners following a VML injury. Results analysed with a two‐way ANOVA for time (weeks) and group. †Statistically difference from week 1 distance, independent of group, *P* < 0.0001. (c) Total distance covered until exhaustion during a treadmill exercise fatigue test completed 3 days prior to study endpoint. Results analysed by one‐way ANOVA. *^,#^Statistically different from uninjured (*) or VML (^#^), *P* < 0.0001. Data reported as means ± SD.

Resistance load on the wheel did not affect the cumulative distance run (Figure 1a, *P* = 0.340) nor the mean daily distance run in VML‐injured mice (*P* = 0.539); however, independent of the resistance group, the mean daily distance run was greater at weeks 4–6 compared to week 1 (Figure 1b, 4.0 ± 2.7 vs. 1.1 ± 0.7 km/day, *P* < 0.001). Results from previous studies that reported as little as 1.5 km/day of wheel running produced muscle adaptations (Call et al., 2010; Warren et al., 2007). An endpoint exhaustive treadmill test was used in this study to validate whole‐body aerobic adaptation. There was a significant effect (*P* < 0.0001) with both VML‐HR and VML‐LR covering a greater distance compared to uninjured and VML (Figure 1c).

### 3.1 Effect of resistance wheel running on physical characteristics of body and muscle mass following VML injury

There were significant effects for terminal body mass, gastrocnemius muscle mass and gastrocnemius mass normalized to body mass (Table 1, *P* ≤ 0.009). Notably, all VML‐injured mice had significantly less gastrocnemius muscle mass compared to uninjured mice, and VML‐HR runners had ∼12% greater muscle mass compared to VML. When gastrocnemius muscle mass was normalized to body mass, the VML‐HR runners were no longer statistically different from uninjured.

**TABLE 1:** Descriptive characteristics.

	Uninjured (*n* = 8)	VML untreated (*n* = 12)	VML–LR (*n* = 8)	VML–HR (*n* = 8)	One‐way ANOVA *P*‐value
Body mass (g)	30.9 ± 1.40^AB^	31.02 ± 1.95^A^	28.83 ± 1.05^B^	29.27 ± 1.51^AB^	0.009
Gastrocnemius mass (mg)	181.95 ± 10.9^A^	133.4 ± 22.6^C^	136.8 ± 15.8^BC^	149.3 ± 12.7^B^	<0.0001
Gastrocnemius mass/body mass (mg/g)	5.88 ± 0.21^A^	4.29 ± 0.68^C^	4.73 ± 0.44^BC^	5.11 ± 0.50^AB^	<0.0001
Muscle mass ratio (injured/uninjured)	1.00 ± 0.06^A^	0.73 ± 0.11^C^	0.80 ± 0.06^BC^	0.88 ± 0.06^B^	<0.0001

Data analysed by one‐way ANOVA, presented as means ± SD. Groups that share a letter are not statistically different.

### 3.2 Effect of resistance wheel running on in vivo muscle contractility following VML injury

**FIGURE 2:**
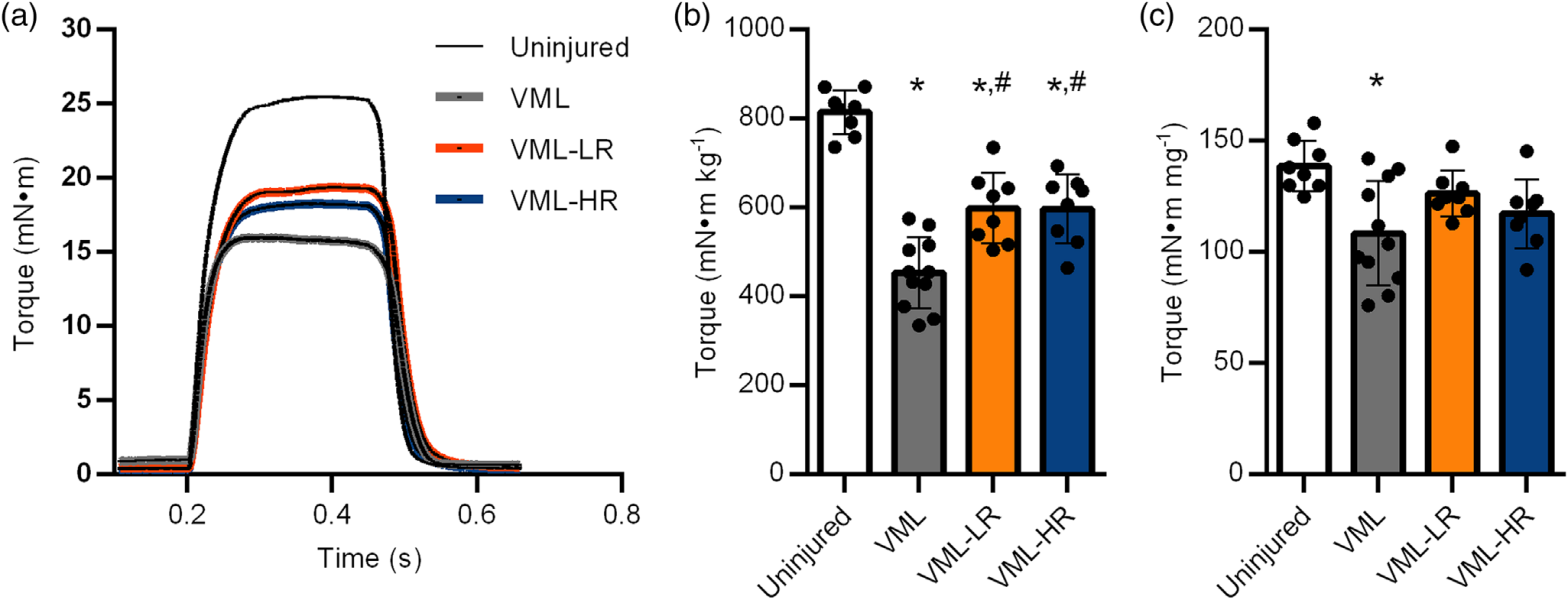
Effect of resistance wheel running on peak isometric torque after VML injury. (a) Representative torque–time tracings. (b) Peak isometric torque normalized to body mass. Data analysed by one‐way ANOVA. *^,#^Statistically different from uninjured (*) or VML (^#^), *P* < 0.0001. (c) Peak isometric torque normalized by gastrocnemius muscle mass. Data analysed by one‐way ANOVA. *Statistically different from uninjured, *P* = 0.004. Data are means ± SD.

Two in vivo tests, peak isometric torque and isokinetic torque, were performed at the study endpoint to evaluate adaptations in muscle strength. There was a significant effect for peak isometric torque normalized by body mass (*P* < 0.0001) as well as peak isometric torque normalized by muscle mass (*P* = 0.004), a marker of muscle quality. All VML‐injured mice had significantly lower peak isometric torque normalized to body mass compared to uninjured; however, VML‐HR and VML‐LR runners had ∼32% greater strength compared to VML (Figure 2a). VML mice had 22% lower peak isometric torque normalized to gastrocnemius muscle mass compared to uninjured, and the wheel running cohorts were not statistically different from uninjured or VML (Figure 2b).

Individual peak isometric torque–time tracings were analysed to determine whether wheel running affected properties indicative of how fast the plantar flexor muscle group contracted and relaxed. There were significant effects on the rate of torque development (*P* < 0.001) and the rate of torque relaxation (*P* < 0.001). The rate of torque development was greatest in uninjured (575 ± 135 mN·m/s) compared to all VML‐injured groups (VML: 357 ± 125; VML‐LR: 376 ± 53; VML‐HR: 367 ± 37 mN·m/s). The rate of torque relaxation was greatest in uninjured (636 ± 78 mN·m/s) compared to all VML‐injured groups; however, VML‐LR and VML‐HR runners had a faster rate of relaxation (454 ± 71 and 495 ± 64 mN·m/s, respectively) compared to VML (261 ± 126 mN·m/s).

**FIGURE 3:**
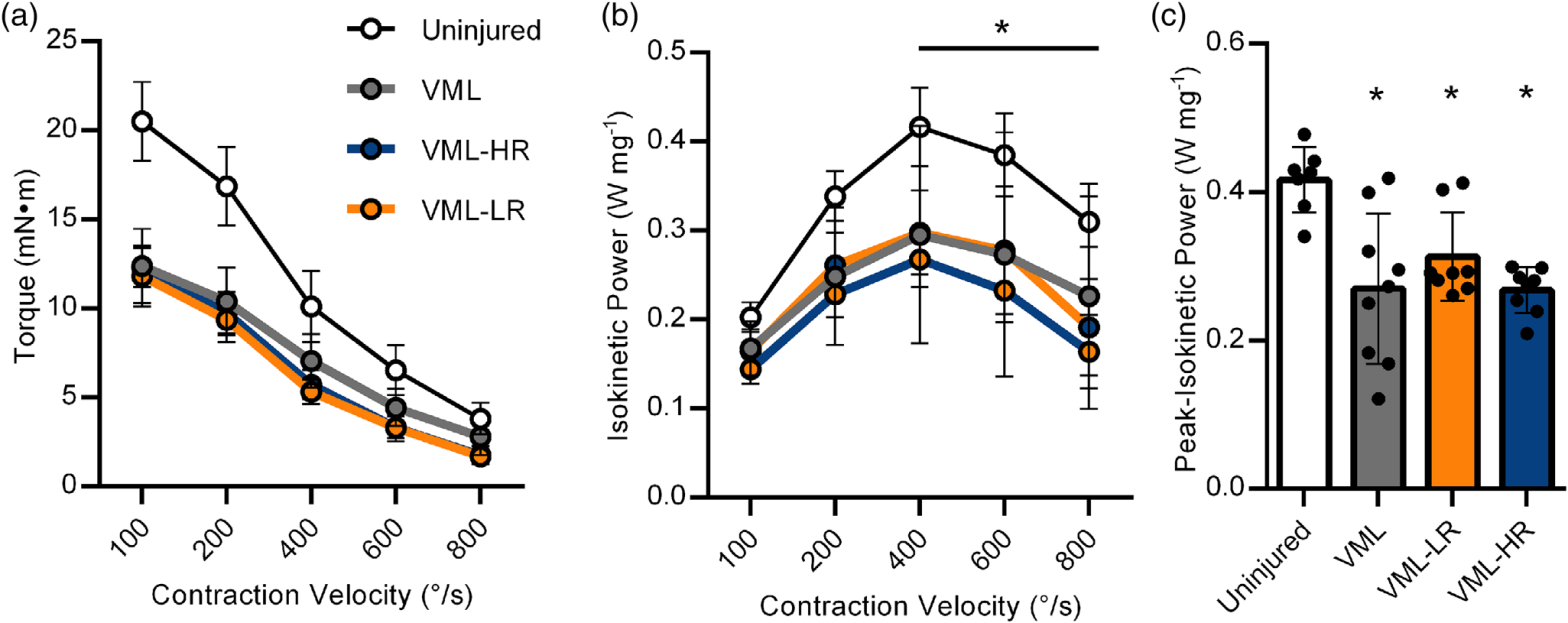
Effect of resistance wheel running on concentric torque–velocity relationship after a VML injury. (a) Concentric torque–contractile velocity relationships. (b) Concentric torque converted to power and normalized by gastrocnemius muscle mass. Data analysed by two‐way ANOVA for contractile velocity and group. *Uninjured greater than VML, VML‐HR, VML‐LR at 400°/s, 600°/s and 800°/velocities, *P* < 0.0001. (c) Peak isokinetic power normalized by gastrocnemius mass. Data analysed by one‐way ANOVA. *Statistically different from uninjured, *P* = 0.005. Data are means ± SD.

During ambulation, rarely are muscles performing peak isometric contractions, and plantarflexion torque required for forward motion changes depending on velocity of movement and load being moved (Baltgalvis et al., 2012). To determine whether wheel running altered plantarflexion contraction velocity or power, concentric torque was tested at different angular velocities. Overall, there was a decrease in concentric torque with increasing contractile velocity (Figure 3a). Isokinetic power was calculated from the concentric contraction data and there was a significant interaction between group and contraction velocity (*P* < 0.0001) for isokinetic power normalized by gastrocnemius muscle mass. Uninjured mice had greater isokinetic power normalized by muscle mass at 400°/s, 600°/s and 800°/s contractile velocities compared to all VML‐injured groups (Figure 3b). Furthermore, there was a significant effect for peak isokinetic power normalized by muscle mass (*P* = 0.005) with uninjured mice having ∼78% greater peak isokinetic power compared to all VML‐injured groups (Figure 3c).

**FIGURE 4:**
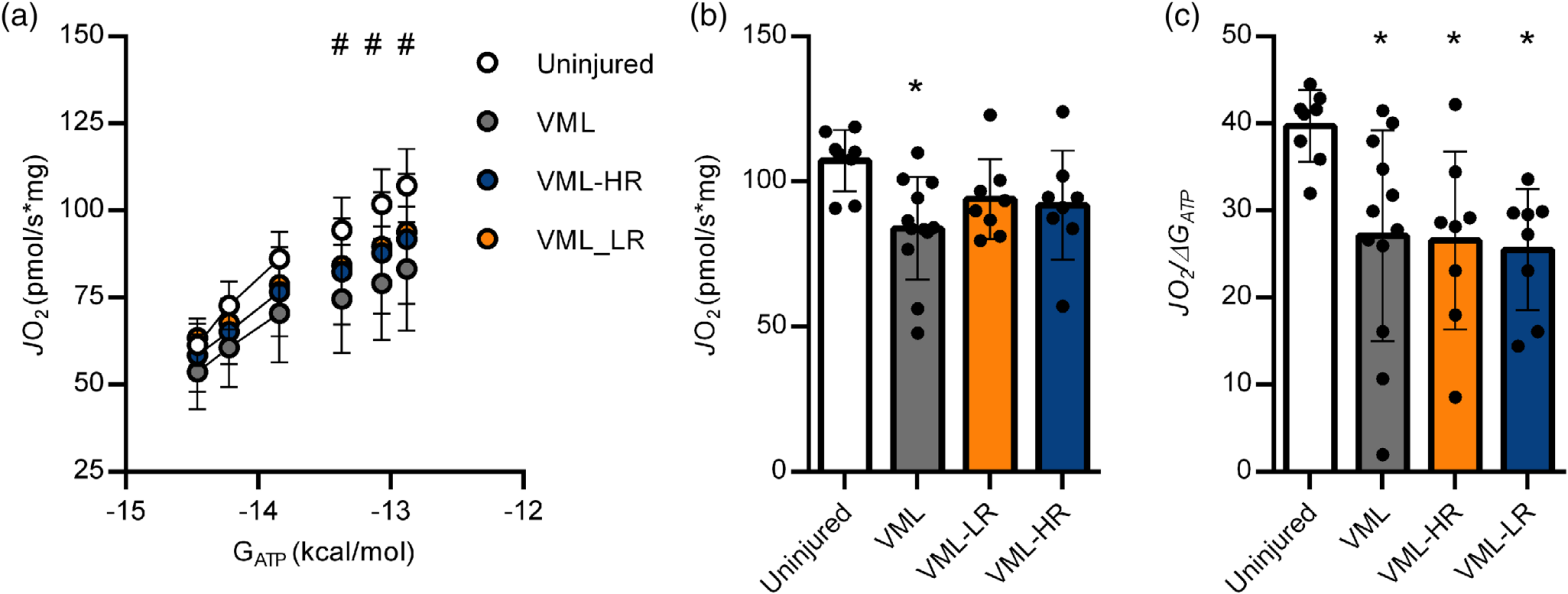
Effect of resistance wheel running on permeabilized fibre bundle mitochondrial respiration after a VML injury. (a) Relationship between clamped ATP free energy states (*G*_ATP_) and permeabilized fibre bundle mitochondrial oxygen consumption ($J_{O_{2}}$). Data analysed by two‐way ANOVA for clamped *G*_ATP_ and group. ^#^Uninjured greater than VML, *P* = 0.0002. (b) Maximal permeabilized fibre bundle mitochondrial $J_{O_{2}}$. Data analysed by one‐way ANOVA. *Statistically different from uninjured, *P* = 0.025. (c) Electron conductance calculated as the slope of the linear phase of the CK clamp (connected symbols in panel *A*). Data analysed by Kruskal–‐Wallis test; *statistically different from uninjured, *P* = 0.004. Data are means ± SD.

The most robust adaptation to aerobic exercise training is an increase in cellular oxidative capacity, often measured experimentally through cellular respiration and mitochondrial content. In agreement with a previous report, uninjured and VML‐injured permeabilized fibre bundle mitochondria were sensitive to the bioenergetic CK clamp approach (McFalin et al., 2023), and independent of injury $J_{O_{2}}$ diminished with more negative ∆*G*_ATP_ during the CK clamp test (Figure 4a). There was a significant interaction between the group and clamped ∆*G*_ATP_ during the $J_{O_{2}}$ testing (*P* = 0.0002). Uninjured $J_{O_{2}}$ was greater than VML at the Gibbs free energy states of −13.84, −13.37 and −13.07, and maximal $J_{O_{2}}$ (∆*G*_ATP_ −12.88) was 22% greater in uninjured compared to VML (Figure 4b). Electron conductance, the ease of electron flow through the electron transport chain, was evaluated by calculating the slope of the linear phase of the CK clamp (∆*G*_ATP_ −13.84, −14.22, −14.46). There was a significant effect for electron conductance (*P* = 0.004), and uninjured conductance was ∼50% greater than all VML‐injured cohorts (Figure 4c).

**FIGURE 5:**
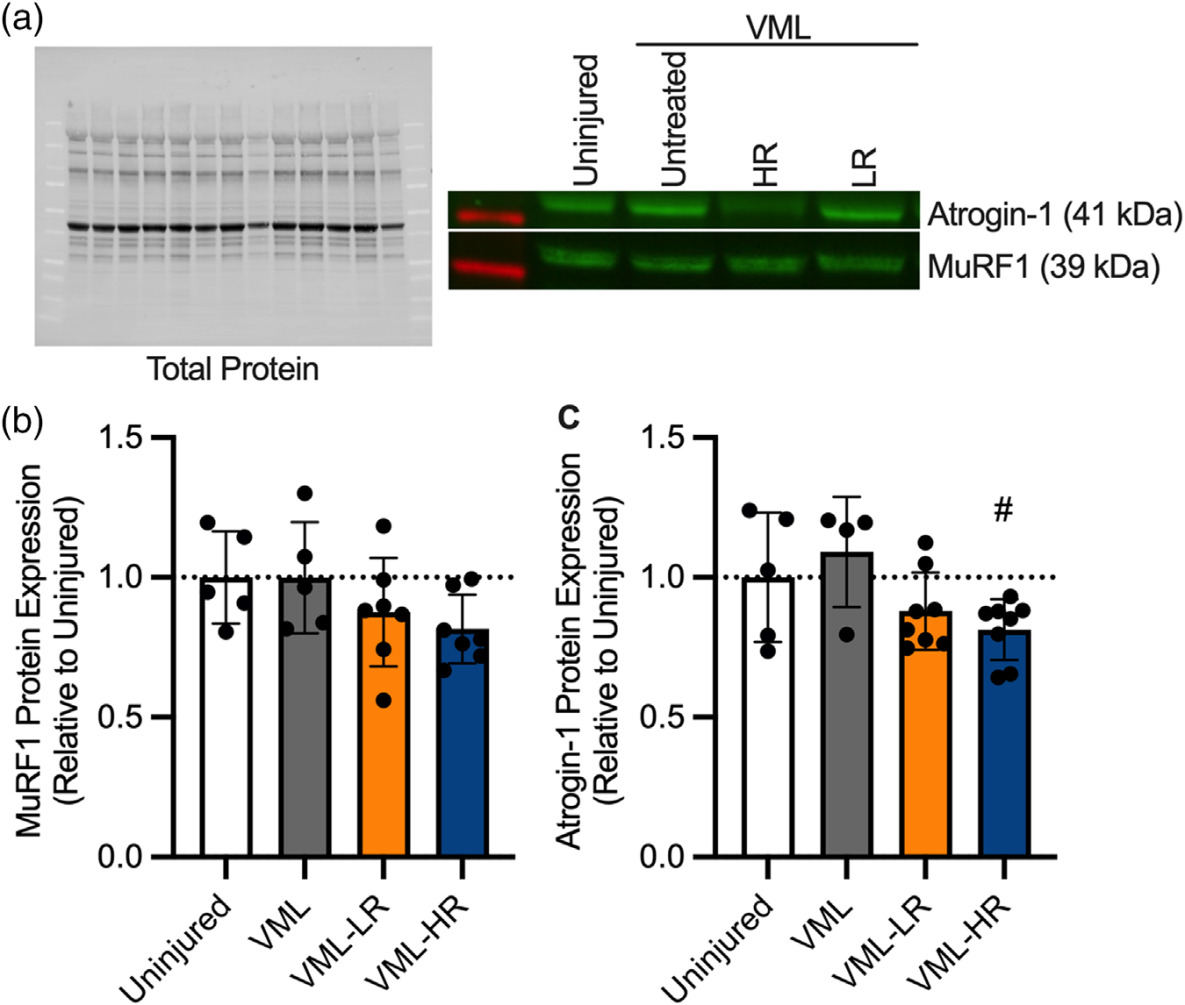
Effect of resistance wheel running on atrophy‐related protein expression. (a) Representative images of total lane protein evaluated using stain‐free imaging and corresponding bands identified at the molecular masses indicated for atrogin‐1 and MuRF1. (b) Relative protein expression of MuRF1 was similar across groups (*P* = 0.193). (c) Atrogin‐1 was significantly reduced when a high‐resistance wheel running regimen was implemented. ^#^Statistically different from VML, *P* = 0.043. Data are means ± SD.

Wheel runners had a modest, yet statistically significant, improvement in muscle strength compared to untreated VML, and particularly in the VML‐HR runners the gains in muscle strength were primarily explained by greater muscle mass. To explore the cellular signalling pathways that can contribute to greater muscle mass, the relative protein contents for hypertrophy‐ and atrophy‐related proteins were examined in the gastrocnemius muscle. Regarding muscle atrophy‐related proteins, there was not a statistically significant effect for MuRF1, an E3 ubiquitin ligase associated with muscle atrophy (Figure 5a,b; *P* = 0.193). There was a significant effect for atrogin‐1, a muscle‐specific F‐box protein also associated with muscle atrophy (Figure 5a,c; *P* = 0.043). The relative protein content of atrogin‐1 was 26% less in VML‐HR runners compared to untreated VML.

**FIGURE 6:**
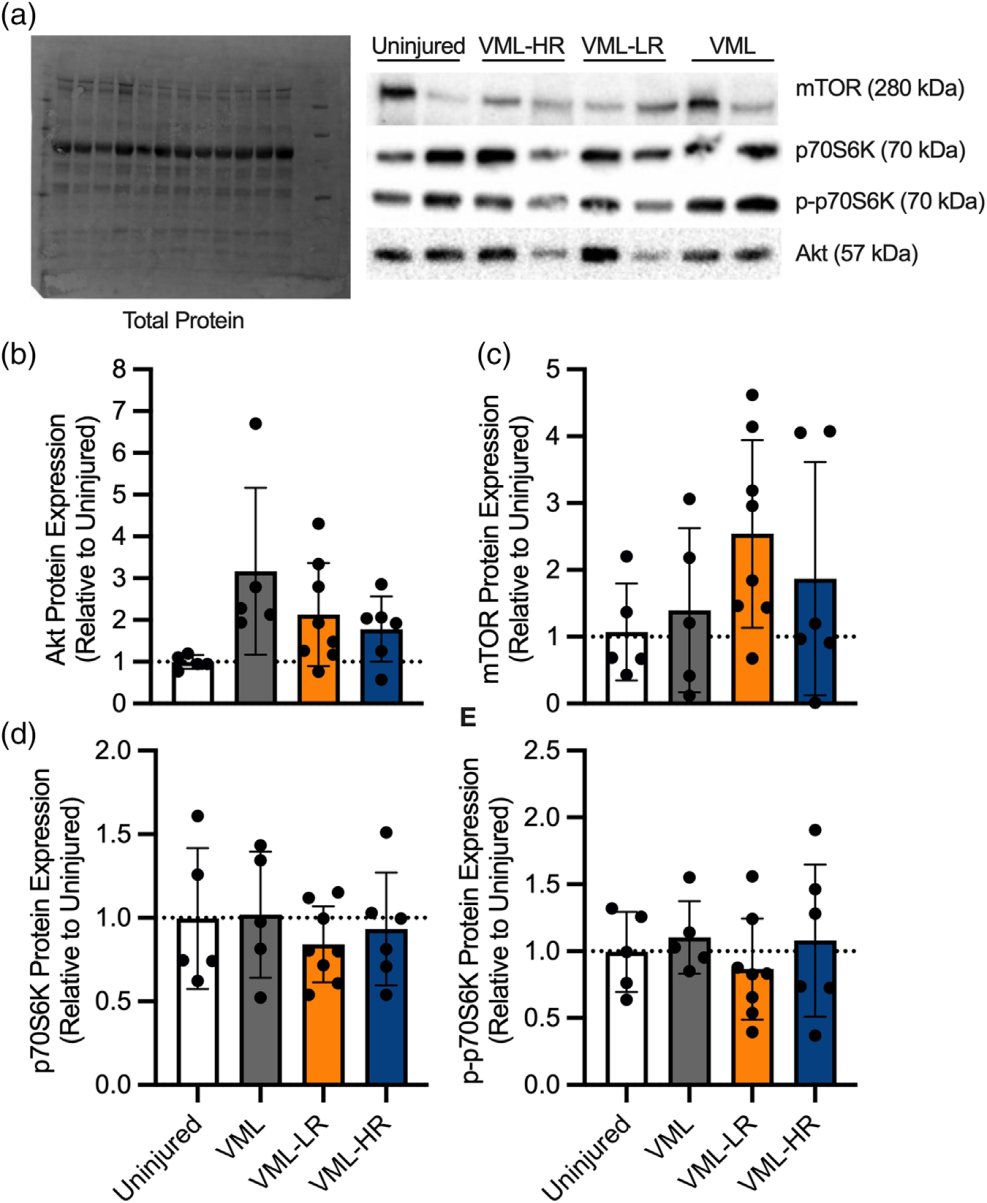
Effect of resistance wheel running on protein synthesis‐related protein expression. (a) Representative images of total lane protein evaluated using stain‐free imaging and corresponding bands identified at the molecular weights indicated for all markers. (b–e) Relative protein expression of Akt, mTOR, p70S6K and p‐p70S6K was similar across groups, *P* ≥ 0.070. Data are means ± SD.

The content of proteins associated with muscle protein synthesis, Akt, mTOR, p70S6K, and phospho‐p70S6K, was also evaluated. There were no statistically significant effects for any relative protein contents (Figure 6; *P* ≥ 0.070). Finally, there was no significant effect for the relative protein content of the transcription factor PGC‐1α (relative expression 0.85 ± 0.24; *P* = 0.330), which typically trends with mitochondrial content and can be activated with aerobic training such as voluntary wheel running.

## 4 DISCUSSION

The main findings revealed resistance wheel running improved peak isometric torque, in part due to higher gastrocnemius mass, but did not impact isokinetic torque, molecular signalling, or mitochondrial capacity or biogenesis, suggesting resistance wheel running alone has a modest benefit in the VML‐injured muscle. Following VML injury, evidence suggests the skeletal muscle lacks a positive structural and functional adaptative response to physical rehabilitation, unlike skeletal muscle following a minor injury (e.g., strain). Although the physiological mechanisms for this diminished response are not completely understood, it is likely due to a host of factors (Greising et al., 2017), including pathological fibrosis (Corona, Rivera et al., 2018; Garg et al., 2015; Hoffman et al., 2022), chronic inflammation (Corona, Rivera et al., 2018; Hurtgen et al., 2016; Larouche et al., 2022), secondary denervation (Corona, Flanagan et al., 2018; Sorensen et al., 2021) maladaptive neurotrophic activity (Hoffman et al., 2023), and reduced metabolic gene and protein expression and mitochondrial function (Aurora et al., 2015; Greising et al., 2018; Southern et al., 2019), among other factors. Preclinical physical rehabilitation regimens to date have varied in the timing of initiation following injury (e.g., early or delayed), duration (e.g., 4, 8, 16 weeks), mode (e.g., running, electrical stimulation, passive range of motion), frequency and volume (Greising et al., 2019). Further, research supports better outcomes when early mobilization is implemented following injuries ranging from acute muscle strain to critical illness (Bayer et al., 2017; Miranda Rocha et al., 2017). A regimen that incorporates resistance running in the early stages (i.e., 3 days) following VML injury may help promote concomitant adaptations to skeletal muscle hypertrophy, and an increased endurance capacity following VML due to the nature of the exercise (i.e., resistance and aerobic). Therefore, this work evaluated low‐ and high‐resistance running as a physical rehabilitative intervention to improve functional recovery of the muscle remaining following VML injury. Our primary finding is that resistance wheel running can produce positive adaptations in VML‐injured muscle chronically, as demonstrated by higher gastrocnemius muscle mass and peak isometric torque than when the muscle is left untreated.

Normally following a minor skeletal muscle injury, degeneration occurs within the first 3 days, followed by regeneration and remodelling of the muscle, resulting in the muscle recovering full functionality (e.g., peak isometric torque). In contrast, after VML injury, the muscle remaining lacks an adequate regenerative response and demonstrates chronic functional deficits (Aguilar et al., 2018; Garg et al., 2015; Larouche et al., 2023). With the implementation of resistance running, a regimen expected to stimulate hypertrophy and thus improve force production in healthy skeletal muscle or following a minor muscle injury, peak isometric torque normalized by body mass was enhanced above the level of untreated mice. However, this difference was negated when peak isometric torque was normalized by gastrocnemius muscle mass. Similarly, isokinetic concentric torque (i.e., dynamic muscle strength evaluation method), a more translational and functionally relevant measure than isometric force (i.e., static muscle strength assessment method) (Mendoza & Miller, 2014; Wang et al., 2020), was not improved with the addition of resistance running. These observations may be supported by evidence indicating excessive collagen deposition, as well as alterations in collagen composition and organization, is a contributing factor to increased muscle stiffness and passive torque following VML injury (Corona et al., 2020, 2016; Garg et al., 2014; Greising et al., 2018; Hoffman et al., 2022). In turn, a stiff muscle may have greater difficulty performing isokinetic tasks (dynamic muscle action), resulting in worse functional recovery in isokinetic contractions as compared to isometric (static muscle action). Not surprisingly, resistance running does not directly target the prevention of fibrotic tissue accumulation. Instead, it is possible that treatment with an anti‐fibrotic pharmacological agent could be used in combination with resistance running to decrease fibrotic tissue accumulation in the early phase following VML injury, with progressively overloaded resistance running to improve the functional aspects of the muscle remaining chronically. These speculations warrant additional investigation.

To determine contributing factors to the discrepancies observed between torque normalized by body mass and muscle mass, muscle was evaluated in relation to signalling involved in protein synthesis and breakdown. Following VML injury, there is a frank loss of muscle fibres due to the injury itself (i.e., primary) and later within the remaining muscle because of the sequelae of the injury (i.e., secondary) (Corona et al., 2016). In healthy skeletal muscle, resistance exercise imparts an overload on the skeletal muscle that initiates an intracellular signalling cascade within the muscle, including activation of the PI3K/Akt/mTOR/p70S6K pathway, stimulating muscle protein synthesis and resulting skeletal muscle hypertrophy. Concurrently, this pathway works to limit atrophic signalling through the inhibition of the FoxO family of transcription factors by Akt. Although there was no evidence of protein synthesis‐related alterations with running chronically after VML, it is possible that these pathways were upregulated earlier in the physical rehabilitation regimen, resulting in a higher gastrocnemius mass. In fact, hypertrophy can be observed in as few as 2 weeks with resistance training, necessitating early signalling of protein synthesis. Therefore, future investigations are needed to investigate early (e.g., 2 weeks post‐VML) atrophic, hypertrophic and metabolic signalling in response to a resistance running regimen following VML injury to understand the molecular signalling time course. High‐resistance running lowered atrogin‐1 expression in the muscle remaining, indicating reduced activation of atrophy‐related pathways with this regimen. In accordance with this observation, gastrocnemius mass normalized by body mass was higher with high‐resistance running, possibly suggesting survival of remaining muscle fibres with or without an increase in muscle fibre size or number. However, this remains unclear and warrants additional studies. Finally, it is noteworthy that possible interferences in molecular signalling and resultant muscle adaptations may have occurred with a concurrent resistance and endurance training regimen. For example, endurance training signalling (e.g., AMPK activation) may interfere with mTOR activation (Hawley, 2009). However, given resistance running increased gastrocnemius mass and peak isometric torque above untreated, interference was likely negligible during the resistance running regimen.

In addition to stimulating skeletal muscle hypertrophy, resistance running promotes metabolic, and thus mitochondrial, adaptations within healthy skeletal muscle. Specifically, aerobic training (e.g., running) activates multiple downstream intracellular signalling factors, including the master regulator of mitochondrial biogenesis PGC‐1α, to elicit increases in mitochondrial volume and oxygen consumption at the muscle level. However, resistance running failed to upregulate PGC‐1α expression and increase permeabilized fibre bundle mitochondrial oxygen consumption beyond the levels observed with no treatment. Observations corroborate those reported previously in response to various physical rehabilitation stimuli (Aurora et al., 2014; Basten et al., 2023; Greising et al., 2018; Southern et al., 2019), indicating a blunted response of the muscle to physical rehabilitation following VML injury. However, improvements in mitochondrial oxygen consumption are observed when PGC‐1α is overexpressed (Southern et al., 2019). Although PGC‐1α cannot be directly administered clinically, this research indicates that the muscle can modestly adapt metabolically given the appropriate stimulus. It is possible that an intervention which incorporates resistance running in combination with a pharmacological mitochondrial activator, such as a β_2_‐adrenergic receptor agonist (McFalin et al., 2022; Raymond‐Pope et al., 2023), could improve the capacity of the muscle to respond metabolically.

Daily and cumulative running distance discrepancies between this study and the literature were expected considering the VML injury and its effect on gait (Dienes et al., 2019). Moreover, voluntary wheel running distance is highly variable in mice and can be influenced by the running wheel orientation (Manzanares et al., 2018), the type of inbred mouse strain (Ghosh et al., 2010) (Lerman et al., 2002), whether the mouse is a wild mouse, in nature, or a laboratory mouse (Meijer & Robbers, 2014), or if the mouse has been genetically bred for wheel running (Dumke et al., 2001). Herein, the running wheels were modified with external loads added to the outer wheel circumference as reported in Murach et al. (2020). However, in contrast to that study, the loads herein were not progressively increased over time and were instead applied (1 and 6 g) for the study duration. The daily running discrepancies notwithstanding, which can exceed 12 km/day for healthy C57BL/6J mice, the adaptations associated with the wheel running protocol used herein are supported by two important results. First, daily wheel running distance was similar to ranges reported previously following VML injury (i.e., ∼1–3.5 km/day) and increased over time, peaking during weeks 4–6 relative to week 1, a similar trend to previous reports (Aurora et al., 2014; Corona et al., 2013; Southern et al., 2017; Washington et al., 2021). Second, wheel runners had twice the endurance capacity on the exhaustive treadmill test compared to sedentary mice. Pathophysiologies related to VML (e.g., denervation, loss of total fibre numbers, impaired molecular signalling and metabolic homeostasis) are likely the reason for why muscle‐specific adaptations were not detected, and overcoming this obstacle is a primary challenge for the field.

### 4.1 Conclusions

Despite unsuccessful attempts to recover muscle function chronically after VML injury with physical rehabilitation, it is important to note that physical rehabilitation strategies could be effective if the appropriate type, timing, intensity, overload, frequency and volume are determined. Our observations suggest that intensity as a physical rehabilitation variable should be considered when developing and validating physical rehabilitation strategies to address functional deficits in VML‐injured muscle. It is possible that a progressive overloading resistance running protocol could be implemented in conjunction with pharmacological adjuvant therapies to bolster skeletal muscle hypertrophy, while also mitigating fibrotic depositions and/or activating mitochondrial biogenesis. Indeed, well‐choreographed manipulation of the skeletal muscle niche through pharmaceutical and chronic physical rehabilitation may synergistically promote functional skeletal muscle recovery following VML. Ongoing and future research is needed to determine the molecular signalling pathways affected by these physical rehabilitation strategies, and more importantly, why skeletal muscles remain resilient to positive metabolic adaptations following traumatic muscle injury.

## AUTHOR CONTRIBUTIONS

Conception or design of the work: Sarah M. Greising and Jarrod A. Call. Acquisition, analysis, or interpretation of data for the work: Albino G. Schifino, Christiana J. Raymond‐Pope, Junwon Heo, Jennifer McFaline‐Figueroa, Jarrod A. Call, and Sarah M. Greising. Drafting of the work or revising it critically for important intellectual content: Albino G. Schifino, Christiana J. Raymond‐Pope, Jarrod A. Call, and Sarah M. Greising. All authors approved the final version of the manuscript. All authors agree to be accountable for all aspects of the work in ensuring that questions related to the accuracy or integrity of any part of the work are appropriately investigated and resolved. All persons designated as authors qualify for authorship, and all those who qualify for authorship are listed.

## CONFLICT OF INTEREST

The authors declare that they have no competing interests.

## Supporting information

Statistical Summary Document

## Data Availability

The datasets used and/or analysed during the current study are primarily presented in the current manuscript and are available from the corresponding author on request.
